# Supplementary material for: Cluster Analysis of Medicinal Plants and Targets Based on Multipartite Network
Source: Biomolecules. 2021 Apr 8;11(4):546. doi: 10.3390/biom11040546 (PMC8068312; doi:10.3390/biom11040546)
Supplement: Supplementary file 1 [file biomolecules-11-00546-s001.pdf]

Cluster Analysis of Medicinal Plants and Targets Based on Multipartite Network

Namgil Lee[1,2], Hojin Yoo[2], Heejung Yang[2,3,*]

[1]Department of Information Statistics, Kangwon National University, Gangwondaehak-gil 1, Chuncheon, Gangwon 24341, Republic of Korea

[2]Bionsight, Inc., Gangwondaehak-gil 1, Chuncheon, Gangwon 24341, Republic of Korea

[3]Laboratory of Natural Products Chemistry, Department of Pharmacy, Kangwon National University, Gangwondaehak-gil 1, Chuncheon, Gangwon 24341, Republic of Korea

*** Correspondence:**
Heejung Yang
heejyang@kangwon.ac.kr

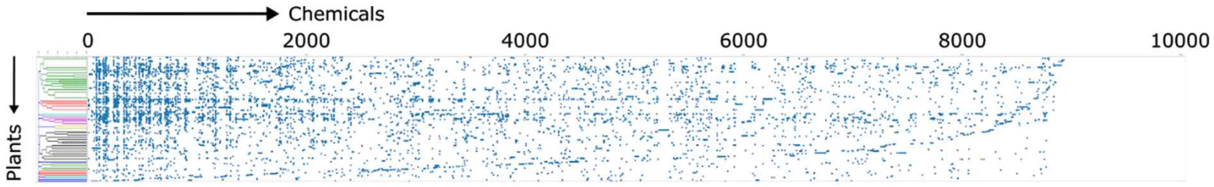

Figure S1. Visualization of non-zero values of the data matrix for the plants and chemicals, where the rows correspond to the plants and the columns correspond to the chemicals. The rows were permuted according to the order determined by the hierarchical clustering of the plants.

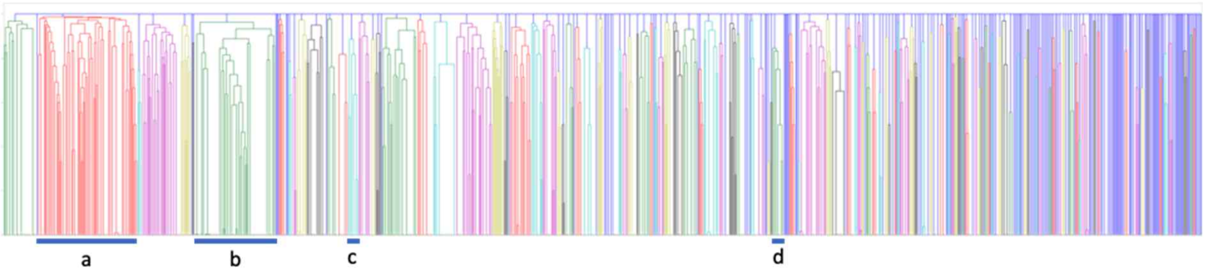

Figure S2. The whole dendrogram for clustering of plants obtained by the hierarchical clustering analysis. **a-d**: the dendrograms of **Figures 2A, 2C**, **2E** and **2H**, respectively.

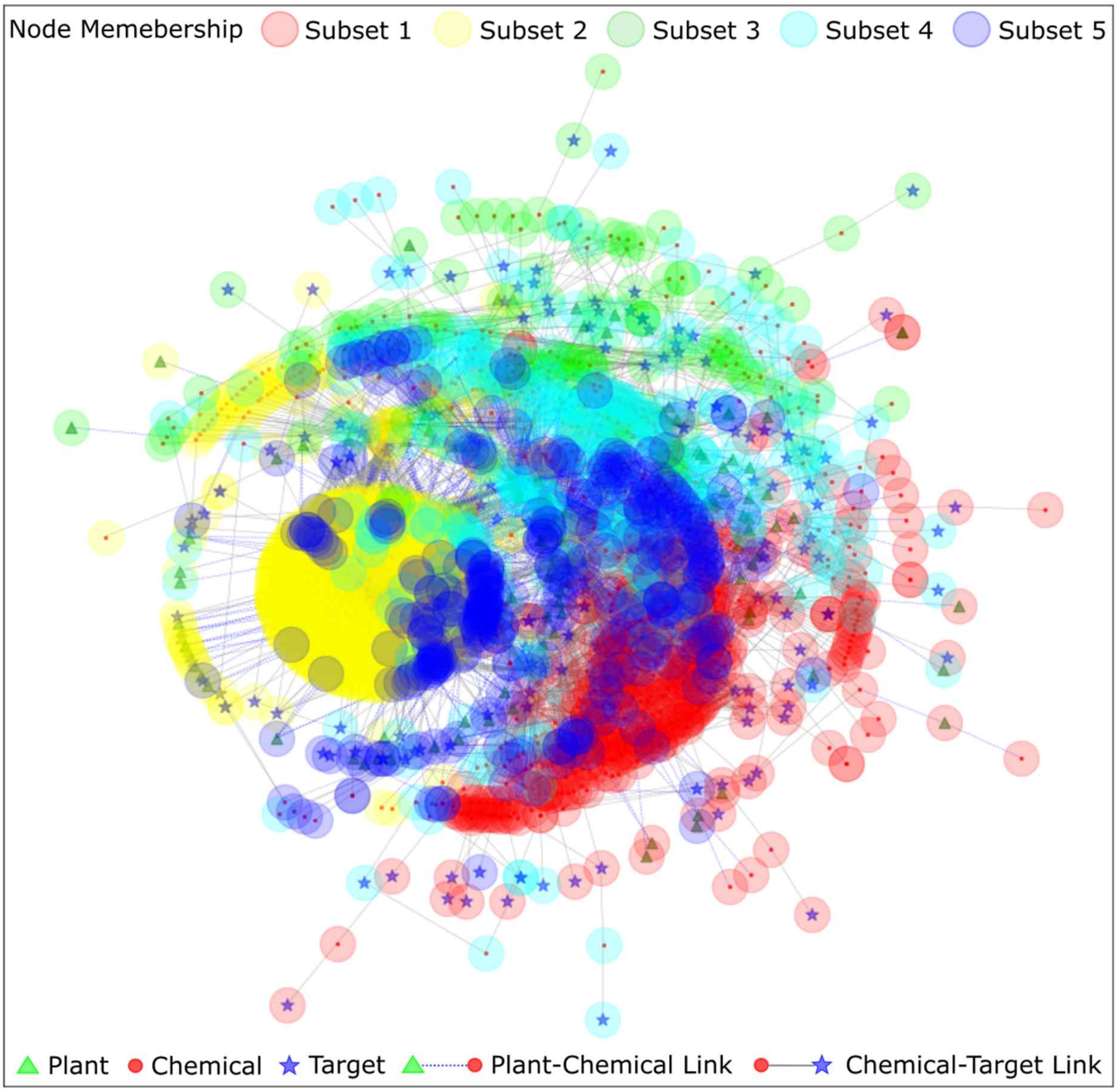
Node Memebership
Subset 1
Subset 2
Subset 3
Subset 4
Subset 5
Plant
Chemical
Target
Plant-Chemical Link
Chemical-Target Link
(A)

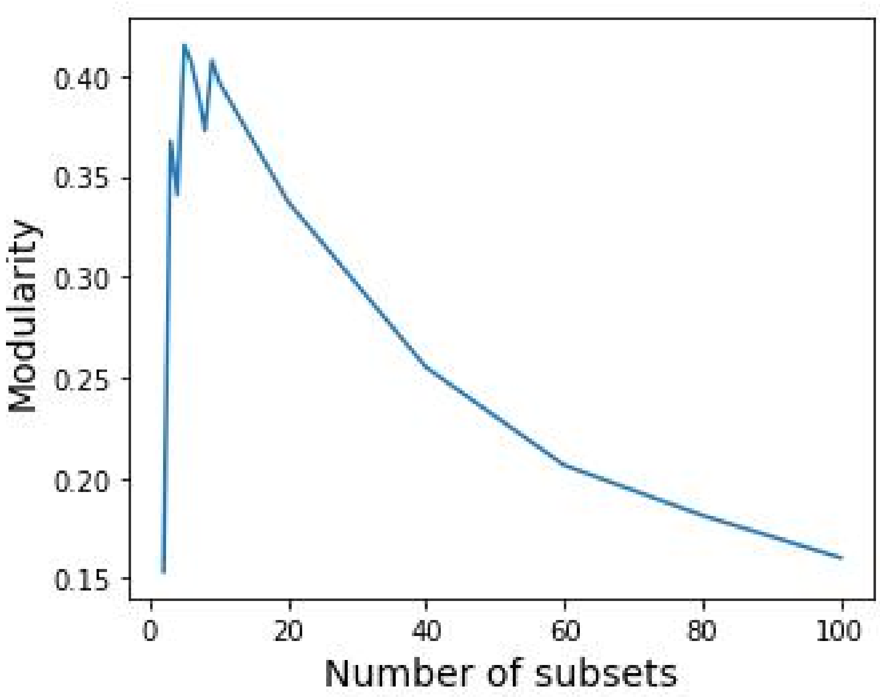

(B)

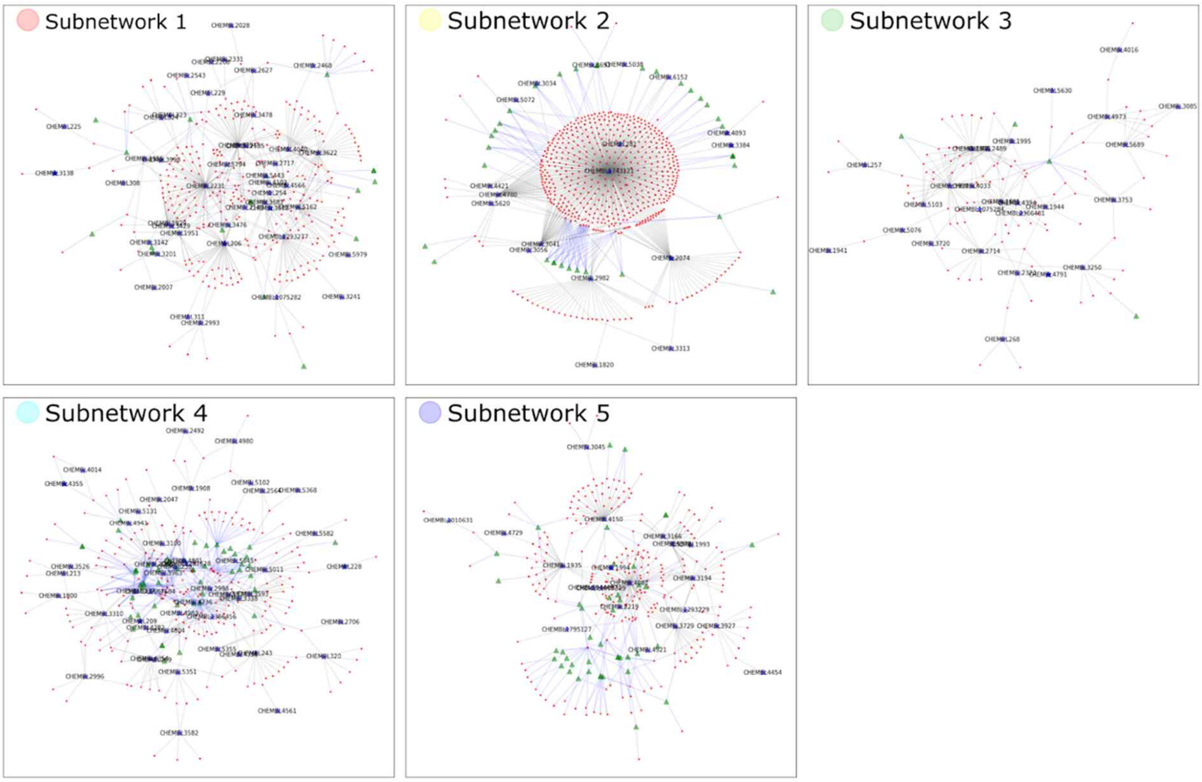

(C)

Figure S3. Visualization of the results of the spectral co-clustering. **(A)** Spectral co-clustering of the whole network. **(B)** The modularity values for the determination of the number of clusters for the spectral co-clustering algorithm. **(C)** The subnetworks induced by each of the

five subsets of nodes obtained by the spectral co-clustering. The 10% of the whole nodes were randomly selected for visualization.

Table S1. The structural properties of the whole network and the five subnetworks obtained by the spectral co-clustering algorithm.

Network	Number of Nodes				Number of Edges			Diameter	Average Bipartite Clustering Coefficient	Degree Assortativity
	Np	Nc	Nt	N	Epc	Ect	E			
Whole	1138	10051	1224	12413	34549	100290	134839	5	0.0946	-0.1954
Sub 1	276	3459	124	3859	3915	29493	33408	4	0.2116	-0.3936
Sub 2	327	2538	136	3001	3946	13456	17402	5	0.1595	-0.3672
Sub 3	34	676	177	887	205	5491	5696	5	0.1544	-0.4156
Sub 4	141	1907	361	2409	1564	14118	15682	4	0.1315	-0.3814
Sub 5	360	1471	426	2257	9751	5893	15644	5	0.1436	-0.2290

Np=number of plant nodes, Nc=number of chemical nodes, Nt=number of target nodes, N=total number of nodes, Epc=number of plant-chemical edges, Ect=number of chemical-target edges, E=total number of edges.

Table S2. List of plants illustrated in **Figures 2A** and **2B**.

Plant ID	Scientific Name	Latin Name	Family
**M0000066**	*Euphorbia kansui*	Euphorbiae Kansui Radix	Euphorbiaceae
**M0000069**	*Citrus suavissima*	Citri Suavissimae Exocarpium	Rutaceae
**M0000072**	*Glycyrrhiza uralensis*	Glycyrrhizae Praeparatus Pulvis	Leguminosae
**M0000075**	*Glycyrrhiza inflata*	Glycyrrhizae Radix	Leguminosae
**M0000079**	*Glycyrrhiza inflata*	Glycyrrhizae Resina	Leguminosae
**M0000080**	*Glycyrrhiza uralensis*	Glycyrrhizae Radicii Virgula	Leguminosae
**M0000088**	*Pinellia ternata*	Pinelliae Praeparatum cum Zingiberis	Araceae
**M0000110**	*Dianthus superbus*	Dianthi Herba	Caryophyllaceae
**M0000127**	*Zizyphus jujuba var. inermis*	Zizyphi Fructus	Rhamnaceae
**M0000216**	*Melia azedarach*	Meliae Cortex	Meliaceae
**M0000222**	*Melia azedarach*	Meliae Fructus	Meliaceae
**M0000224**	*Melia azed-arach var. japonica*	Meliae Cortex	Meliaceae
**M0000262**	*Cucumis melo*	Melo Pediculus	Cucurbitaceae
**M0000264**	*Sorghum vulgare*	Sorghi Acetum	Gramineae
**M0000265**	*Hordeum vulgare*	Hordei Acetum	Gramineae
**M0000334**	*Hordeum vulgare*	Hordei Fructus	Gramineae
**M0000567**	*Raphanus sativus*	Raphani Radix	Cruciferae
**M0000581**	*Kochia scoparia*	Kochiae Fructus	Chenopodiaceae
**M0000607**	*Strobilenthes flaccidifolia*	Strobilenthis Sedimentum	Acanthaceae
**M0000646**	*Raphanus sativus*	Raphani Semen	Cruciferae
**M0000771**	*Echinops latifolius*	Rhapontici Radix	Compositae
**M0000816**	*Lophatherum gracile*	Lophatheri Herba	Gramineae
**M0000854**	*Hordeum vulgare*	Hordei Semen	Gramineae
**M0000855**	*Hordeum vulgare*	Hordei Semen Pulvis	Gramineae
**M0000856**	*Hordeum vulgare*	Hordei Novella	Gramineae
**M0000877**	*Zizyphus vulgaris var. spinosus*	Jujubae Fructus	Rhamnaceae
**M0000878**	*Zizyphus jujuba var. inermis*	Jujubae Fructus	Rhamnaceae
**M0000990**	*Eucommia ulmoides*	Eucommiae Cortex	Eucommiaceae
**M0001001**	*Juncus effusus*	Junci Medulla	Juncaceae

**M0001022**	*Aristolochia contorta*	Aristolochiae Fructus	Aristolochiaceae
**M0001046**	*Calvatia gigantea*	Lasiosphaera Seu Calvatia	Rosacean
**M0001069**	*Portulaca oleracea*	Portulacea Herba	Portulacaceae
**M0001089**	*Vitex trifolia*	Viticis Fructus	Verbenaceae
**M0001108**	*Liriope platyphylla*	Liriopes Radix	Liliaceae
**M0001114**	*Hordeum vulgare var. hexastichon*	Hordei Fructus Germiniatus	Gramineae
**M0001115**	*Hordeum vulgare*	Hordei Fructus Germinatus	Gramineae
**M0001189**	*Aralia elata*	Araliae Cortex Lignum seu Radicis	Araliaceae
**M0001234**	*Ficus carica*	Fici Fructus	Moraceae
**M0001303**	*Pinellia pedatisecta*	Pinelliae Rhizoma	Araceae
**M0001305**	*Pinellia ternata*	Pinelliae Rhizoma Fermentata	Araceae
**M0001309**	*Eleocharis dulcis*	Eleocharitis Tuber	Cyperaceae
**M0001357**	*vulgaris Lamarck var. spinosus*	Zizyphi Spina	Rhamnaceae
**M0001377**	*Ampelopsis japonica*	Ampelopsis Radix	Vitaceae
**M0001523**	*Ficus pumila*	Fici Pumilae Caulis	Moraceae
**M0001541**	*Psoralea corylifolia*	Psoraleae Fructus	Leguminosae
**M0001581**	*Ailanthus altissima|Ailanthus glandulosa*	Ailanthi Herba	Simaroubaceae
**M0001628**	*Torreya nucifera*	Torreyae Semen	Taxaceae
**M0001629**	*Torreya grandis*	Torreyae Semen	Taxaceae
**M0001663**	*Adenophora triphylla var. japonica*	Adenophorae Radix	Campanulaceae
**M0001710**	*Sambucus javanica*	Sambuci Radix	Caprifoliaceae
**M0001780**	*Gardenia jasminoides*	Gardeniae Fructus	Rubiaceae
**M0001808**	*Morus alba*	Mori Cortex	Moraceae
**M0001829**	*Cynanchum atratum*	Cynanchi Radix	Asclepiadaceae
**M0001836**	*Morus alba*	Mori Favilla	Moraceae
**M0001839**	*Morus alba*	Mori Fructus	Moraceae
**M0001855**	*Morus alba*	Mori Ramulus	Moraceae
**M0001940**	*Dendrobium loddigesii*	Dendrobii Herba	Orchidaceae
**M0002165**	*Cynomorium songaricum*	Cynomorii Herba	Cynomoriaceae
**M0002301**	*Centipeda minima*	Centipedae Herba	Compositae
**M0002470**	*Hordeum vulgare*	Hordei Germinatus	Gramineae

**M0002552**	*Mirabilis jalapa*	Fucus cum Jalapae Tuber	Nyctaginaceae
**M0002555**	*Mirabilis jalapa*	Fucus cum Jalapae Embryo	Nyctaginaceae
**M0002598**	*Acanthopanax sessiliflorus*	Acanthopanacis Cortex	Araliaceae
**M0002661**	*Lindera aggregata*	Linderae Radix	Lauraceae
**M0002913**	*Citrus suavissima*	Citri Suavissimae Fructus	Rutaceae
**M0002915**	*Artemisia anomala|Artemisia viridissima*	Artemisiae Anomalae Herba	Compositae
**M0002923**	*Ulmus macrocarpa*	Ulmi Cortex	Ulmaceae
**M0002991**	*Coix lachryma-jobi var. ma-yeun*	Coicis Radix	Gramineae
**M0002992**	*Coix lachryma-jobi*	Coicis Radix	Gramineae
**M0002993**	*Coix lachryma-jobi var. ma-yeun*	Coicis Semen	Gramineae
**M0002994**	*Coix lachryma-jobi*	Coicis Semen	Gramineae
**M0003064**	*Glycyrrhiza uralensis*	Glycyrrhizae Radix Praeparata	Leguminosae
**M0003088**	*Spirodela polyrrhyza*	Spirodelae Herba	Lemnaceae
**M0003284**	*Tamarix chinensis*	Tamaricis Ramulus	Tamaricaceae
**M0003373**	*Adenophora trachelioides*	Trachelioidis Radix	Campanulaceae
**M0003415**	*Zizyphus jujuba var. inermis*	Zizyphi Radix	Rhamnaceae
**M0003417**	*Zizyphus jujuba var. inermis*	Zizyphi Folium	Rhamnaceae
**M0003492**	*Kochia scoparia*	Kochiae Herba	Chenopodiaceae
**M0003630**	*Euphorbia lathyris*	Euphorbiae Lathyridis Semen	Euphorbiaceae
**M0003670**	*Aristolochia contorta*	Aristolochiae Herba	Aristolochiaceae
**M0003719**	*Cucumis melo*	Melo Fructus	Cucurbitaceae
**M0003720**	*Cucumis melo*	Melo Semen	Cucurbitaceae
**M0003721**	*Cucumis melo*	Melonis Pedicelus	Cucurbitaceae
**M0003731**	*Baphicacanthus cusia*	Indigo Pulverata Levis	Acanthaceae
**M0003752**	*Morus alba*	Mori Radicis Cortex	Moraceae
**M0003786**	*Celosia argentea*	Celosiae Semen	Amaranthaceae
**M0003830**	*Sorghum vulgare*	Sorghi Radix	Gramineae
**M0003831**	*Dichroa febrifuga|Orixa japonica*	Dichroae Arbor Novella	Saxifragaceae
**M0003858**	*Sorghum vulgare*	Sorghi Semen	Gramineae
**M0003887**	*Gardenia jasminoides*	Gardeniae Semen	Rubiaceae
**M0003969**	*Psoralea corylifolia*	Psoraleae Semen	Leguminosae

**M0004009**	*Patrinia scabiosaefolia*	Patriniae Radix	Valerianaceae
**M0004073**	*Prunella vulgaris*	Prunellae Spica	Labiatae
**M0004247**	*Cucumis sativus*	Cucumeris Fructus	Cucurbitaceae
**M0004398**	*Cudrania tricuspidata*	Cudraniae Folium	Moraceae

Table S3. List of plants illustrated in **Figures 2C** and **2D**.

Plant ID	Scientific Name	Latin Name	Family
**M0000060**	*Saccharum sinensis*	Sacchari Tuber	Gramineae
**M0000124**	*Caesalpinia sappan|Cercis chinensis*	Fucus cum Caesalpiniae	Leguminosae
**M0000179**	*Areca catechu*	Arecae Semen	Palmae
**M0000227**	*Cannabis sativa*	Vetus Cannabis Solea	Cannabinaceae
**M0000263**	*Oryza sativa*	Oryzae Acetum	Gramineae
**M0000286**	*Oryza sativa*	Oryzae Fructus Germinatus	Gramineae
**M0000557**	*Oryza sativa*	Oryzae Glutinosa Medulla	Gramineae
**M0000559**	*Oryza sativa*	Oryzae Radix seu Rhizoma	Gramineae
**M0000564**	*Oryza sativa*	Oryzae Semen	Gramineae
**M0000565**	*Oryza sativa*	Alcohol cum Oryzae Semen	Gramineae
**M0000803**	*Santalum album*	Santali Albae Lignum	Santalaceae
**M0000821**	*Angelica sinensis*	Angelicae Gigantis Radix	Umbelliferae
**M0000842**	*Glycine max*	Glycinis Semen	Leguminosae
**M0000844**	*Glycine max*	Glycinis Semen Germinatum	Leguminosae
**M0000846**	*Glycine max*	Glycine Semen Germinatum	Leguminosae
**M0000850**	*Cannabis sativa*	Cannabis Herba	Cannabinaceae
**M0000852**	*Cannabis sativa*	Cannabis Semen	Cannabinaceae
**M0000862**	*Areca catechu*	Arecae Pericarpium	Palmae
**M0000864**	*Allium sativum*	Allii Bulbus	Liliaceae
**M0000913**	*Oryza sativa*	Oryzae Semen Germinatus	Gramineae
**M0000938**	*Benincasa cerifera|Benincasa hispida*	Benincasae Fructus	Cucurbitaceae
**M0000939**	*Benincasa cerifera|Benincasa hispida*	Benincasae Caulis	Cucurbitaceae
**M0000940**	*Benincasa cerifera|Benincasa hispida*	Benincasae Folium	Cucurbitaceae
**M0000984**	*Glycine max*	Alcohol cum Glycinis Semen	Leguminosae
**M0000986**	*Glycine max*	Glycinis Semen Ptisanari	Leguminosae
**M0000992**	*Glycine max*	Glycinis Semen	Leguminosae
**M0001013**	*Agaricus campestris*	Agaricus Polyporus	Agaricaceae
**M0001014**	*Cannabis sativa*	Cannabis Radix	Cannabinaceae

**M0001058**	*Cannabis sativa*	Cannabis Folium	Cannabinaceae
**M0001062**	*Cannabis sativa*	Cannabis Fructus	Cannabinaceae
**M0001191**	*Auricularia auricula-judae*	Auriculariae Polyporus	Auriculariaceae
**M0001197**	*Auricularia auricula-judae*	Auriculariae Polyporus	Auriculariaceae
**M0001359**	*Santalum album*	Santali Albi Lignum	Santalaceae
**M0001418**	*Oryza sativa*	Oryzae Semen Pulvus	Gramineae
**M0001422**	*Saccharum sinensis*	Saccharum Alba	Gramineae
**M0001473**	*Oryza sativa*	Oryzae Ptisanari	Gramineae
**M0001540**	*Oryza sativa*	Alcohol Orizae cum Medico Fermento	Gramineae
**M0001639**	*Saccharum sinensis*	Saccharum Glacialis	Gramineae
**M0001659**	*Saccharum sinensis*	Saccharum	Gramineae
**M0001901**	*Scutellaria baicalensis*	Scutellariae Radix	Labiatae
**M0002086**	*Caesalpinia sappan|Cercis chinensis*	Sappan Lignum	Anacardiaceae
**M0002150**	*Tricholoma matsutake*	Tricholomae Polyporus	Tricholomataceae
**M0002185**	*Polygonum hydropiper*	Polygoni Hydropiper Herba	Polygonaceae
**M0002221**	*Benincasa cerifera|Benincasa hispida*	Benincasae Fructus	Cucurbitaceae
**M0002476**	*Glycine max*	Glycinis Semen	Leguminosae
**M0002477**	*Glycine max*	Glycinis Testa	Leguminosae
**M0002568**	*Oryza sativa*	Vinum Ferri	Gramineae
**M0002573**	*Glycine max*	Faba cum Sale Fermentoque Praeparata	Leguminosae
**M0002721**	*Pisum sativum*	Pisi Flos	Leguminosae
**M0002742**	*Polygonum hydropiper*	Polygoni Fructus	Polygonaceae
**M0002806**	*Cyathula capitata*	Achyranthis Radix	Amaranthaceae
**M0002807**	*Cyathula officinalis*	Achyranthis Radix	Amaranthaceae
**M0002810**	*Achyranthes bidentata*	Achyranthis Radix	Amaranthaceae
**M0002870**	*Glycine max*	Glycine Semen Nigrae	Leguminosae
**M0003033**	*Panax ginseng*	Ginseng Radix	Araliaceae
**M0003035**	*Panax ginseng*	Ginseng Nodus	Araliaceae
**M0003066**	*Sagittaria sagittifolia*	Sagittariae Tuber	Alismataceae
**M0003235**	*Oryza sativa*	Oryzae Testa	Gramineae
**M0003380**	*Capsella bursa-pastoris*	Capsellae Herba	Cruciferae

**M0003381**	*Capsella bursa-pastoris*	Capsellae Semen	Cruciferae
**M0003559**	*Oryza sativa*	Arida Oryza Distillata	Gramineae
**M0003622**	*Angelica sinensis*	Angelicae Sinensis Radix	Umbelliferae
**M0003764**	*Glycine max*	Liquor Salsus ex Faba	Leguminosae
**M0003765**	*Oryza sativa*	Purum Vinum Oryzae	Gramineae
**M0003853**	*Oryza sativa*	Vinum in Primo Vere	Gramineae
**M0003864**	*Oryza sativa*	Arida Oryza Praeparatum	Gramineae
**M0004061**	*Ricinus communis*	Ricini Folium	Euphorbiaceae
**M0004062**	*Ricinus communis*	Ricini Semen	Euphorbiaceae
**M0004267**	*Coriandrum sativum*	Coriandri Herba cum Radix	Umbelliferae
**M0004268**	*Coriandrum sativum*	Coriandri Herba cum Radix	Umbelliferae
**M0004269**	*Coriandrum sativum*	Coriandri Fructus	Umbelliferae
**M0004270**	*Glycine max*	Glycinis Semen Praeparatum	Leguminosae
**M0004286**	*Piper nigrum*	Pireris Nigri Fructus	Piperaceae
**M0004303**	*Alpinia galanga*	Galangae Fructus	Zingiberaceae
**M0004460**	*Glycine max*	Glycinis Semen Nigra	Leguminosae
**M0004463**	*Saccharum sinensis*	Saccharum Nigrum	Gramineae
**M0004468**	*Glycine max*	Glycinis Nigra Testa	Leguminosae

Table S4. List of plants illustrated in **Figures 2E** and **2F**.

Plant ID	Scientific Name	Latin Name	Family
**M0000806**	*Phellodendron amurense*	Phellodendri Caulis	Rutaceae
**M0002558**	*Corydalis ternata*	Corydalis (Tuber) Rhizoma	Papaveraceae
**M0003700**	*Coptis japonica*	Coptidis Rhizoma	Ranunculaceae
**M0004375**	*Coptis deltoidea*	Coptidis Rhizoma	Ranunculaceae
**M0004376**	*Coptis teetoides*	Coptidis Rhizoma	Ranunculaceae
**M0004377**	*Coptis japonica var. dissecta*	Coptidis Rhizoma	Ranunculaceae
**M0004379**	*Coptis japonica*	Alcohol cum Coptidis Rhizoma	Ranunculaceae
**M0004380**	*Coptis chinensis*	Alcohol cum Coptidis Rhizoma	Ranunculaceae
**M0004387**	*Phellodendron chinense*	Phellodendri Cortex	Rutaceae
**M0004394**	*Phellodendron amurense*	Phellodendri Radix	Rutaceae

Table S5. List of plants illustrated in **Figures 2G** and **2H**.

Plant ID	Scientific Name	Latin Name	Family
**M0000112**	*Prunus humilis*	Pruni Humilis Semen	Rosaceae
**M0000501**	*Apium graveolens var. dulce*	Apii Herba	Umbelliferae
**M0001048**	*Lasiosphaera nipponica*	Lasiosphaera Seu Calvatia	Lycoperdaceae
**M0002838**	*Prunus humilis*	Pruni Cortex	Rosaceae
**M0002840**	*Prunus humilis*	Pruni Folium	Rosaceae
**M0003141**	*Amaranthus mangostanus*	Amaranthi Herba	Amaranthaceae
**M0003628**	*Prunus humilis*	Pruni Lignum	Rosaceae
**M0003833**	*Allium fistulosum*	Allii Radix	Liliaceae
**M0003836**	*Allium fistulosum*	Allii Fistulosi Succus	Liliaceae
**M0003852**	*Ailanthus altissima|Ailanthus glandulosa*	Ailanthi Folium	Simaroubaceae
**M0004209**	*Amaranthus mangostanus*	Amaranthi Semen	Amaranthaceae

Table S6. List of chemicals which are targeted on phosphodiesterase 10A in **Figure 3A**.

Chemical ID	Chemical Name	inchikey	Chemical Class				
			Kingdom	Superclass	Class	Subclass	Direct_parent
**1570**	1-(2'-Hydroxyethyl)-4-methoxy-beta-carboline	XAKXPUZLFGDHAO-UHFFFAOYSA-N	Organic compounds	Alkaloids and derivatives	Harmala alkaloids	None	Harmala alkaloids
**5411**	1-Hydroxycanthin-6-one	LWYFITNQEPSUDK-UHFFFAOYSA-N	Organic compounds	Alkaloids and derivatives	Indolonaphthyridine alkaloids	None	Indolonaphthyridine alkaloids
**1574**	1-Methoxycanthin-6-one	LEPXKGXTXIACRO-UHFFFAOYSA-N	Organic compounds	Alkaloids and derivatives	Indolonaphthyridine alkaloids	None	Indolonaphthyridine alkaloids
**17289**	1-Hydroxycanthin-6-one; Me ether	LEPXKGXTXIACRO-UHFFFAOYSA-N	Organic compounds	Alkaloids and derivatives	Indolonaphthyridine alkaloids	None	Indolonaphthyridine alkaloids
**537**	Canthin-6-one	ZERVJPYNQLONEK-UHFFFAOYSA-N	Organic compounds	Alkaloids and derivatives	Indolonaphthyridine alkaloids	None	Indolonaphthyridine alkaloids
**629**	2-Ethyl-3-methylpyrazine	LNIMMWYNSBZESE-UHFFFAOYSA-N	Organic compounds	Organoheterocyclic compounds	Diazines	Pyrazines	Pyrazines
**1558**	2-Hydroxycanthin-6-one	BDCOKQCMZJCYDY-UHFFFAOYSA-N	Organic compounds	Alkaloids and derivatives	Indolonaphthyridine alkaloids	None	Indolonaphthyridine alkaloids
**631**	2-Methylpyrazine	CAWHJQAVHZEVTJ-UHFFFAOYSA-N	Organic compounds	Organoheterocyclic compounds	Diazines	Pyrazines	Pyrazines
**632**	2-Methoxy-3-methylpyrazine	VKJIAEQRKBQLLA-UHFFFAOYSA-N	Organic compounds	Organoheterocyclic compounds	Diazines	Pyrazines	Methoxypyrazines
**1561**	5-Hydroxycanthin-6-one	HOTCTNQHGRFRLK-UHFFFAOYSA-N	Organic compounds	Alkaloids and derivatives	Indolonaphthyridine alkaloids	None	Indolonaphthyridine alkaloids
**630**	2-Ethylpyrazine	KVFIJIWMDBAGDP-UHFFFAOYSA-N	Organic compounds	Organoheterocyclic compounds	Diazines	Pyrazines	Pyrazines
**1564**	5-Hydroxymethylcanthin-6-one	NGLFCPGASUBUBB-UHFFFAOYSA-N	Organic compounds	Alkaloids and derivatives	Indolonaphthyridine alkaloids	None	Indolonaphthyridine alkaloids

Table S7. List of chemicals linked with the plant, *Morus alba* and the target, tyrosinase.

Chemical ID	Chemical Name	inchikey	Chemical Class				
			Kingdom	Superclass	Class	Subclass	Direct_parent
**7808**	Mulberrofuran A	MQYYTNPXQXSQGM-CAOOACKPSA-N	Organic compounds	Phenylpropanoids and polyketides	2-arylbenzofuran flavonoids	None	2-arylbenzofuran flavonoids
**6657**	Morachalcone A	NXBYIJSAISXPKJ-WEVVVXLNSA-N	Organic compounds	Phenylpropanoids and polyketides	Linear 1,3-diarylpropanoids	Chalcones and dihydrochalcones	3-prenylated chalcones
**7809**	Resorcinol	GHMLBKRAJCXXBS-UHFFFAOYSA-N	Organic compounds	Benzenoids	Phenols	Benzenediols	Resorcinols
**7810**	Oxydihydroresveratrol	IEOZKGCYMAJAHS-UHFFFAOYSA-N	Organic compounds	Phenylpropanoids and polyketides	Stilbenes	None	Stilbenes
**7811**	Dihydroresveratrol	HITJFUSPLYBJPE-UHFFFAOYSA-N	Organic compounds	Phenylpropanoids and polyketides	Stilbenes	None	Stilbenes
**7812**	Oxyresveratrol (E-form)	PDHAOJSHSJQANO-OWOJBTEDSA-N	Organic compounds	Phenylpropanoids and polyketides	Stilbenes	None	Stilbenes
**7813**	beta-Resorcylaldehyde	IUNJCFABHJZSKB-UHFFFAOYSA-N	Organic compounds	Organic oxygen compounds	Organooxygen compounds	Carbonyl compounds	Hydroxybenzaldehydes
**788**	Albafuran A	KGOOVUKZICPAIZ-FRKPEAEDSA-N	Organic compounds	Phenylpropanoids and polyketides	2-arylbenzofuran flavonoids	None	2-arylbenzofuran flavonoids
**789**	Albafuran B	ODASNNUHHLRPEL-FRKPEAEDSA-N	Organic compounds	Phenylpropanoids and polyketides	2-arylbenzofuran flavonoids	None	2-arylbenzofuran flavonoids
**791**	Albanol A	MJJWBJFYYRAYKU-UHFFFAOYSA-N	Organic compounds	Phenylpropanoids and polyketides	2-arylbenzofuran flavonoids	None	2-arylbenzofuran flavonoids
**792**	5,7-Dihydroxychromone	NYCXYKOXLNBYID-UHFFFAOYSA-N	Organic compounds	Organoheterocyclic compounds	Benzopyrans	1-benzopyrans	Chromones
**793**	Dimoracin	GKHRLTCUMXVTAV-UHFFFAOYSA-N	Organic compounds	Phenylpropanoids and polyketides	2-arylbenzofuran flavonoids	None	2-arylbenzofuran flavonoids
**17947**	1-(2,4-Dihydroxyphenyl)-2-(3,5-dihydroxyphenyl)ethylene; 3'-O-beta-D-Glucopyranoside	GGQVPULXXVQLRT-OWOJBTEDSA-N	Organic compounds	Phenylpropanoids and polyketides	Stilbenes	Stilbene glycosides	Stilbene glycosides
**796**	Moracin C	ZTGHWUWBQNCCOH-UHFFFAOYSA-N	Organic compounds	Phenylpropanoids and polyketides	2-arylbenzofuran flavonoids	None	2-arylbenzofuran flavonoids
**797**	Mulberrofuran F	SCNZCLDHJJSZBK-UHFFFAOYSA-N	Organic compounds	Phenylpropanoids and polyketides	2-arylbenzofuran flavonoids	None	2-arylbenzofuran flavonoids
**17948**	cis-Mulberroside A	HPSWAEGGWLOOKT-UPHRSURJSA-N	Organic compounds	Phenylpropanoids and polyketides	Stilbenes	Stilbene glycosides	Stilbene glycosides
**799**	Mulberrofuran P	WZXFDNVWWQJWRC-UHFFFAOYSA-N	Organic compounds	Phenylpropanoids and polyketides	2-arylbenzofuran flavonoids	None	2-arylbenzofuran flavonoids

**7337**	Mulberranol	FMKONXHUEWRDEL-UHFFFAOYSA-N	Organic compounds	Phenylpropanoids and polyketides	Flavonoids	Flavones	6-prenylated flavones
**8623**	Resveratrol	LUKBXSAWLPMMSZ-OWOJBTEDSA-N	Organic compounds	Phenylpropanoids and polyketides	Stilbenes	None	Stilbenes
**8369**	Broussonin A	MSNVBURPCQDLEP-UHFFFAOYSA-N	Organic compounds	Phenylpropanoids and polyketides	Linear 1,3-diarylpropanoids	Cinnamylphenols	Cinnamylphenols
**8370**	Broussonin B	CJJJQWAYMRTLJT-UHFFFAOYSA-N	Organic compounds	Phenylpropanoids and polyketides	Linear 1,3-diarylpropanoids	Cinnamylphenols	Cinnamylphenols
**823**	Moracin M	LHPRYOJTASOZGJ-UHFFFAOYSA-N	Organic compounds	Phenylpropanoids and polyketides	2-arylbenzofuran flavonoids	None	2-arylbenzofuran flavonoids
**8887**	Kuwanon Y	YYUHPJKWIHNMSV-NSCUHMNNSA-N	Organic compounds	Phenylpropanoids and polyketides	Diarylheptanoids	Linear diarylheptanoids	Linear diarylheptanoids
**1215**	omega-Hydroxymoracin N	GPKLNIVEGYWQJZ-BIIKFXOESA-N	Organic compounds	Phenylpropanoids and polyketides	2-arylbenzofuran flavonoids	None	2-arylbenzofuran flavonoids
**1225**	Moracin N	WBSCSIABHGPAMC-UHFFFAOYSA-N	Organic compounds	Phenylpropanoids and polyketides	2-arylbenzofuran flavonoids	None	2-arylbenzofuran flavonoids
**10575**	Dihydrooxyresveratrol	IEOZKGCYMAJAHS-UHFFFAOYSA-N	Organic compounds	Phenylpropanoids and polyketides	Stilbenes	None	Stilbenes
**6617**	Mulberrin	UWQYBLOHTQWSQD-UHFFFAOYSA-N	Organic compounds	Phenylpropanoids and polyketides	Flavonoids	Flavones	8-prenylated flavones
**6619**	Kuwanon A	DBUNRZUFILGKHP-UHFFFAOYSA-N	Organic compounds	Phenylpropanoids and polyketides	Flavonoids	Flavones	3-prenylated flavones
**6238**	Chrysanthemin	RKWHWFONKJEUEF-UHFFFAOYSA-O	Organic compounds	Phenylpropanoids and polyketides	Flavonoids	Flavonoid glycosides	Anthocyanidin-3-O-glycosides
**6624**	Kuwanon G	APPXYONGBIXGRO-UHFFFAOYSA-N	Organic compounds	Phenylpropanoids and polyketides	Flavonoids	Flavones	8-prenylated flavones
**6625**	Kuwanon H	DKBPTKFKCCNXNH-UHFFFAOYSA-N	Organic compounds	Phenylpropanoids and polyketides	Flavonoids	Flavones	8-prenylated flavones
**6626**	Kuwanon I	KBAPHKOHTBBCTO-AWNIVKPZSA-N	Organic compounds	Phenylpropanoids and polyketides	Diarylheptanoids	Linear diarylheptanoids	Linear diarylheptanoids
**6627**	Kuwanon J	KBAPHKOHTBBCTO-AWNIVKPZSA-N	Organic compounds	Phenylpropanoids and polyketides	Diarylheptanoids	Linear diarylheptanoids	Linear diarylheptanoids
**6628**	Kuwanon R	LSWPUMCBBKEXMW-VIZOYTHASA-N	Organic compounds	Phenylpropanoids and polyketides	Diarylheptanoids	Linear diarylheptanoids	Linear diarylheptanoids
**6629**	Kuwanon Q	WDCSNUYKXLXPBM-OVCLIPMQSA-N	Organic compounds	Phenylpropanoids and polyketides	Diarylheptanoids	Linear diarylheptanoids	Linear diarylheptanoids
**6630**	Kuwanon V	SUQUIVSLHDOSQP-REZTVBANSA-N	Organic compounds	Phenylpropanoids and polyketides	Diarylheptanoids	Linear diarylheptanoids	Linear diarylheptanoids
**6631**	Kuwanon K	FEAIYAOTVUYWQZ-UHFFFAOYSA-N	Organic compounds	Phenylpropanoids and polyketides	Flavonoids	Flavones	3'-prenylated flavones
**6633**	Albafuran C	SEUPIEHHWMMMQG-UHFFFAOYSA-N	Organic compounds	Phenylpropanoids and polyketides	2-arylbenzofuran flavonoids	None	2-arylbenzofuran flavonoids

**6636**	Morusinol	AFOKZNPZDXHDHD-UHFFFAOYSA-N	Organic compounds	Phenylpropanoids and polyketides	Flavonoids	Flavones	3-prenylated flavones
**6637**	Mulberrofuran E	RQIKMRKKKIMUNB-UHFFFAOYSA-N	Organic compounds	Phenylpropanoids and polyketides	2-arylbenzofuran flavonoids	None	2-arylbenzofuran flavonoids
**6638**	Mulberrofuran T	XMXZFZDVDCIFKB-UHFFFAOYSA-N	Organic compounds	Phenylpropanoids and polyketides	2-arylbenzofuran flavonoids	None	2-arylbenzofuran flavonoids
**6639**	Mulberrofuran C	WTGKDESIYCVAOP-UHFFFAOYSA-N	Organic compounds	Phenylpropanoids and polyketides	2-arylbenzofuran flavonoids	None	2-arylbenzofuran flavonoids
**6641**	Sanggenon E	NJQZTHTXKPCAHD-UHFFFAOYSA-N	Organic compounds	Phenylpropanoids and polyketides	Stilbenes	None	Stilbenes
**6643**	Sanggenon P	CUJJTBMGUHNKPO-UHFFFAOYSA-N	Organic compounds	Phenylpropanoids and polyketides	Diarylheptanoids	Linear diarylheptanoids	Linear diarylheptanoids
**6644**	Chalcomoracin	SEHVRKPXIDOTRX-UHFFFAOYSA-N	Organic compounds	Phenylpropanoids and polyketides	2-arylbenzofuran flavonoids	None	2-arylbenzofuran flavonoids
**6646**	Morusin	XFFOMNJIDRDDLQ-UHFFFAOYSA-N	Organic compounds	Phenylpropanoids and polyketides	Flavonoids	Flavones	3-prenylated flavones
**6647**	Cyclomulberrin	SYFDWXWLRGHYAJ-UHFFFAOYSA-N	Organic compounds	Phenylpropanoids and polyketides	Flavonoids	Pyranoflavonoids	Pyranoflavonoids
**6648**	Moracenin D	PYXAHGLTSLDDJH-UHFFFAOYSA-N	Organic compounds	Phenylpropanoids and polyketides	Flavonoids	Flavones	8-prenylated flavones
**7806**	2,4-Dihydroxybenzoic acid ethyl ether	BRDIPNLKURUXCU-UHFFFAOYSA-N	Organic compounds	Benzenoids	Benzene and substituted derivatives	Benzoic acids and derivatives	p-Hydroxybenzoic acid alkyl esters
**7807**	Kuwanol E	HGFWVFTZYRJFRI-AATRIKPKSA-N	Organic compounds	Phenylpropanoids and polyketides	Diarylheptanoids	Linear diarylheptanoids	Linear diarylheptanoids

Table S8. List of chemicals linked with the plants, *Carthamus tinctorius*, *Cudrania tricuspidata*, *Glycyrrhiza inflata* and *Glycyrrhiza uralensis* and the target, tyrosinase.

Chemical ID	Chemical Name	inchikey	Chemical Class				
			Kingdom	Superclass	Class	Subclass	Direct_parent
Carthamus tinctorius							
**7232**	Safflomin C	CCEKPTFNQKNHKZ-XCVCLJGOSA-N	Organic compounds	Phenylpropanoids and polyketides	Diarylheptanoids	Linear diarylheptanoids	Curcuminoids
**6187**	Safflor Yellow B	WBKUQZJRZIYAJL-YDWXAUTNSA-N	None	None	None	None	None
**17516**	PRE	ITBPOPSLMGJTQH-YDWXAUTNSA-N	None	None	None	None	None
**7231**	Safflomin A	NNXHCBKOBDDJFM-ZZXKWVIFSA-N	None	None	None	None	None
**920**	N-(p-Coumaroyl)-5-hydroxytryptamine	WLZPAFGVOWCVMG-FPYGCLRLSA-N	Organic compounds	Organoheterocyclic compounds	Indoles and derivatives	Tryptamines and derivatives	N-acylserotonins
**918**	N-Feruloylserotonin	WGHKJYWENWLOMY-XVNBXDOJSA-N	Organic compounds	Organoheterocyclic compounds	Indoles and derivatives	Tryptamines and derivatives	N-acylserotonins
**17495**	4,4''-Bi(N-4-hydroxycinnamoylserotonin); (E,E)-form	OIUNULHHOJRJBI-IAGONARPSA-N	Organic compounds	Organoheterocyclic compounds	Indoles and derivatives	Tryptamines and derivatives	N-acylserotonins
**17496**	4-[N-(p-Coumaroyl)serotonin-4''-yl]-N-feruloylserotonin	ZPNFTINOYMQICL-PWSZKDBUSA-N	Organic compounds	Organoheterocyclic compounds	Indoles and derivatives	Tryptamines and derivatives	N-acylserotonins
**17497**	4,4''-Bis(N-feruloyl)serotonin	URHRFQQPWDNDQE-ACFHMISVSA-N	Organic compounds	Organoheterocyclic compounds	Indoles and derivatives	Tryptamines and derivatives	N-acylserotonins
**7261**	Safflor yellow A	YKDBQWHZEDSTAI-ZZXKWVIFSA-N	Organic compounds	Phenylpropanoids and polyketides	Cinnamic acids and derivatives	Hydroxycinnamic acids and derivatives	Hydroxycinnamic acids and derivatives
**1502**	N-(p-Coumaroyl)serotonin	WLZPAFGVOWCVMG-FPYGCLRLSA-N	Organic compounds	Organoheterocyclic compounds	Indoles and derivatives	Tryptamines and derivatives	N-acylserotonins
**1503**	N-(p-Coumaroyl)serotonin mono-bera-D-glucopyranoside	LPGWQGDUKIPAME-FPYGCLRLSA-N	Organic compounds	Organic oxygen compounds	Organooxygen compounds	Carbohydrates and carbohydrate conjugates	Phenolic glycosides
Cudrania tricuspidata							
**7808**	Mulberrofuran A	MQYYTNPXQXSQGM-CAOOACKPSA-N	Organic compounds	Phenylpropanoids and polyketides	2-arylbenzofuran flavonoids	None	2-arylbenzofuran flavonoids

**6657**	Morachalcone A	NXBYIJSAISXPKJ-WEVVVXLNSA-N	Organic compounds	Phenylpropanoids and polyketides	Linear 1,3-diarylpropanoids	Chalcones and dihydrochalcones	3-prenylated chalcones
**7810**	Oxydihydroresveratrol	IEOZKGCYMAJAHS-UHFFFAOYSA-N	Organic compounds	Phenylpropanoids and polyketides	Stilbenes	None	Stilbenes
**7811**	Dihydroresveratrol	HITJFUSPLYBJPE-UHFFFAOYSA-N	Organic compounds	Phenylpropanoids and polyketides	Stilbenes	None	Stilbenes
**7812**	Oxyresveratrol (E-form)	PDHAOJSHSJQANO-OWOJBTEDSA-N	Organic compounds	Phenylpropanoids and polyketides	Stilbenes	None	Stilbenes
**7813**	beta-Resorcylaldehyde	IUNJCFABHJZSKB-UHFFFAOYSA-N	Organic compounds	Organic oxygen compounds	Organooxygen compounds	Carbonyl compounds	Hydroxybenzaldehydes
**7809**	Resorcinol	GHMLBKRAJCXXBS-UHFFFAOYSA-N	Organic compounds	Benzenoids	Phenols	Benzenediols	Resorcinols
**788**	Albafuran A	KGOOVUKZICPAIZ-FRKPEAEDSA-N	Organic compounds	Phenylpropanoids and polyketides	2-arylbenzofuran flavonoids	None	2-arylbenzofuran flavonoids
**789**	Albafuran B	ODASNNUHHLRPEL-FRKPEAEDSA-N	Organic compounds	Phenylpropanoids and polyketides	2-arylbenzofuran flavonoids	None	2-arylbenzofuran flavonoids
**791**	Albanol A	MJJWBJFYYRAYKU-UHFFFAOYSA-N	Organic compounds	Phenylpropanoids and polyketides	2-arylbenzofuran flavonoids	None	2-arylbenzofuran flavonoids
**792**	5,7-Dihydroxychromone	NYCXYKOXLNBYID-UHFFFAOYSA-N	Organic compounds	Organoheterocyclic compounds	Benzopyrans	1-benzopyrans	Chromones
**793**	Dimoracin	GKHRLTCUMXVTAV-UHFFFAOYSA-N	Organic compounds	Phenylpropanoids and polyketides	2-arylbenzofuran flavonoids	None	2-arylbenzofuran flavonoids
**17947**	1-(2,4-Dihydroxyphenyl)-2-(3,5-dihydroxyphenyl)ethylene; 3'-O-beta-D-Glucopyranoside	GGQVPULXXVQLRT-OWOJBTEDSA-N	Organic compounds	Phenylpropanoids and polyketides	Stilbenes	Stilbene glycosides	Stilbene glycosides
**17948**	cis-Mulberroside A	HPSWAEGGWLOOKT-UPHRSURJSA-N	Organic compounds	Phenylpropanoids and polyketides	Stilbenes	Stilbene glycosides	Stilbene glycosides

**796**	Moracin C	ZTGHWUWBQNCCOH-UHFFFAOYSA-N	Organic compounds	Phenylpropanoids and polyketides	2-arylbenzofuran flavonoids	None	2-arylbenzofuran flavonoids
**797**	Mulberrofuran F	SCNZCLDHJJSZBK-UHFFFAOYSA-N	Organic compounds	Phenylpropanoids and polyketides	2-arylbenzofuran flavonoids	None	2-arylbenzofuran flavonoids
**799**	Mulberrofuran P	WZXFDNVWWQJWRC-UHFFFAOYSA-N	Organic compounds	Phenylpropanoids and polyketides	2-arylbenzofuran flavonoids	None	2-arylbenzofuran flavonoids
**7337**	Mulberranol	FMKONXHUEWRDEL-UHFFFAOYSA-N	Organic compounds	Phenylpropanoids and polyketides	Flavonoids	Flavones	6-prenylated flavones
**8623**	Resveratrol	LUKBXSAWLPMMSZ-OWOJBTEDSA-N	Organic compounds	Phenylpropanoids and polyketides	Stilbenes	None	Stilbenes
**8369**	Broussonin A	MSNVBURPCQDLEP-UHFFFAOYSA-N	Organic compounds	Phenylpropanoids and polyketides	Linear 1,3-diarylpropanoids	Cinnamylphenols	Cinnamylphenols
**8370**	Broussonin B	CJJJQWAYMRTLJT-UHFFFAOYSA-N	Organic compounds	Phenylpropanoids and polyketides	Linear 1,3-diarylpropanoids	Cinnamylphenols	Cinnamylphenols
**823**	Moracin M	LHPRYOJTASOZGJ-UHFFFAOYSA-N	Organic compounds	Phenylpropanoids and polyketides	2-arylbenzofuran flavonoids	None	2-arylbenzofuran flavonoids
**8887**	Kuwanon Y	YYUHPJKWIHNMSV-NSCUHMNNSA-N	Organic compounds	Phenylpropanoids and polyketides	Diarylheptanoids	Linear diarylheptanoids	Linear diarylheptanoids
**1215**	omega-Hydroxymoracin N	GPKLNIVEGYWQJZ-BIIKFXOESA-N	Organic compounds	Phenylpropanoids and polyketides	2-arylbenzofuran flavonoids	None	2-arylbenzofuran flavonoids
**1225**	Moracin N	WBSCSIABHGPAMC-UHFFFAOYSA-N	Organic compounds	Phenylpropanoids and polyketides	2-arylbenzofuran flavonoids	None	2-arylbenzofuran flavonoids
**10575**	Dihydrooxyresveratrol	IEOZKGCYMAJAHS-UHFFFAOYSA-N	Organic compounds	Phenylpropanoids and polyketides	Stilbenes	None	Stilbenes
**6617**	Mulberrin	UWQYBLOHTQWSQD-UHFFFAOYSA-N	Organic compounds	Phenylpropanoids and polyketides	Flavonoids	Flavones	8-prenylated flavones
**6619**	Kuwanon A	DBUNRZUFILGKHP-UHFFFAOYSA-N	Organic compounds	Phenylpropanoids and polyketides	Flavonoids	Flavones	3-prenylated flavones

**6238**	Chrysanthemin	RKWHWFONKJEUEF-UHFFFAOYSA-O	Organic compounds	Phenylpropanoids and polyketides	Flavonoids	Flavonoid glycosides	Anthocyanidin-3-O-glycosides
**6624**	Kuwanon G	APPXYONGBIXGRO-UHFFFAOYSA-N	Organic compounds	Phenylpropanoids and polyketides	Flavonoids	Flavones	8-prenylated flavones
**6625**	Kuwanon H	DKBPTKFKCCNXNH-UHFFFAOYSA-N	Organic compounds	Phenylpropanoids and polyketides	Flavonoids	Flavones	8-prenylated flavones
**6626**	Kuwanon I	KBAPHKOHTBBCTO-AWNIVKPZSA-N	Organic compounds	Phenylpropanoids and polyketides	Diarylheptanoids	Linear diarylheptanoids	Linear diarylheptanoids
**6627**	Kuwanon J	KBAPHKOHTBBCTO-AWNIVKPZSA-N	Organic compounds	Phenylpropanoids and polyketides	Diarylheptanoids	Linear diarylheptanoids	Linear diarylheptanoids
**6628**	Kuwanon R	LSWPUMCBBKEXMW-VIZOYTHASA-N	Organic compounds	Phenylpropanoids and polyketides	Diarylheptanoids	Linear diarylheptanoids	Linear diarylheptanoids
**6629**	Kuwanon Q	WDCSNUYKXLXPBM-OVCLIPMQSA-N	Organic compounds	Phenylpropanoids and polyketides	Diarylheptanoids	Linear diarylheptanoids	Linear diarylheptanoids
**6630**	Kuwanon V	SUQUIVSLHDOSQP-REZTVBANSA-N	Organic compounds	Phenylpropanoids and polyketides	Diarylheptanoids	Linear diarylheptanoids	Linear diarylheptanoids
**6631**	Kuwanon K	FEAIYAOTVUYWQZ-UHFFFAOYSA-N	Organic compounds	Phenylpropanoids and polyketides	Flavonoids	Flavones	3'-prenylated flavones
**6633**	Albafuran C	SEUPIEHHWMMMQG-UHFFFAOYSA-N	Organic compounds	Phenylpropanoids and polyketides	2-arylbenzofuran flavonoids	None	2-arylbenzofuran flavonoids
**6636**	Morusinol	AFOKZNPZDXHDHD-UHFFFAOYSA-N	Organic compounds	Phenylpropanoids and polyketides	Flavonoids	Flavones	3-prenylated flavones
**6637**	Mulberrofuran E	RQIKMRKKKIMUNB-UHFFFAOYSA-N	Organic compounds	Phenylpropanoids and polyketides	2-arylbenzofuran flavonoids	None	2-arylbenzofuran flavonoids
**6638**	Mulberrofuran T	XMXZFZDVDCIFKB-UHFFFAOYSA-N	Organic compounds	Phenylpropanoids and polyketides	2-arylbenzofuran flavonoids	None	2-arylbenzofuran flavonoids
**6639**	Mulberrofuran C	WTGKDESIYCVAOP-UHFFFAOYSA-N	Organic compounds	Phenylpropanoids and polyketides	2-arylbenzofuran flavonoids	None	2-arylbenzofuran flavonoids

**6641**	Sanggenon E	NJQZTHTXKPCAHD-UHFFFAOYSA-N	Organic compounds	Phenylpropanoids and polyketides	Stilbenes	None	Stilbenes
**6643**	Sanggenon P	CUJJTBMGUHNKPO-UHFFFAOYSA-N	Organic compounds	Phenylpropanoids and polyketides	Diarylheptanoids	Linear diarylheptanoids	Linear diarylheptanoids
**6644**	Chalcomoracin	SEHVRKPXIDOTRX-UHFFFAOYSA-N	Organic compounds	Phenylpropanoids and polyketides	2-arylbenzofuran flavonoids	None	2-arylbenzofuran flavonoids
**6646**	Morusin	XFFOMNJIDRDDLQ-UHFFFAOYSA-N	Organic compounds	Phenylpropanoids and polyketides	Flavonoids	Flavones	3-prenylated flavones
**6647**	Cyclomulberrin	SYFDWXWLRGHYAJ-UHFFFAOYSA-N	Organic compounds	Phenylpropanoids and polyketides	Flavonoids	Pyranoflavonoids	Pyranoflavonoids
**6648**	Moracenin D	PYXAHGLTSLDDJH-UHFFFAOYSA-N	Organic compounds	Phenylpropanoids and polyketides	Flavonoids	Flavones	8-prenylated flavones
**7806**	2,4-Dihydroxybenzoic acid ethyl ether	BRDIPNLKURUXCU-UHFFFAOYSA-N	Organic compounds	Benzenoids	Benzene and substituted derivatives	Benzoic acids and derivatives	p-Hydroxybenzoic acid alkyl esters
**7807**	Kuwanol E	HGFWVFTZYRJFRI-AATRIKPKSA-N	Organic compounds	Phenylpropanoids and polyketides	Diarylheptanoids	Linear diarylheptanoids	Linear diarylheptanoids
Glycyrrhiza inflata							
**17792**	Licochalcone C	WBDNTJSRHDSPSR-KPKJPENVSA-N	Organic compounds	Phenylpropanoids and polyketides	Linear 1,3-diarylpropanoids	Chalcones and dihydrochalcones	Retrochalcones
**7300**	5'-Prenyllicodione	VAWLLIOUAFRMHN-UHFFFAOYSA-N	Organic compounds	Phenylpropanoids and polyketides	Linear 1,3-diarylpropanoids	Chalcones and dihydrochalcones	Retro-dihydrochalcones
**7301**	Glycyrdione A	VDOHBGQSFOWYTB-UHFFFAOYSA-N	Organic compounds	Phenylpropanoids and polyketides	Linear 1,3-diarylpropanoids	Chalcones and dihydrochalcones	Retro-dihydrochalcones
**7303**	Glyinflanin A	VDOHBGQSFOWYTB-UHFFFAOYSA-N	Organic compounds	Phenylpropanoids and polyketides	Linear 1,3-diarylpropanoids	Chalcones and dihydrochalcones	Retro-dihydrochalcones
**7317**	Licochalcone C	WBDNTJSRHDSPSR-KPKJPENVSA-N	Organic compounds	Phenylpropanoids and polyketides	Linear 1,3-diarylpropanoids	Chalcones and dihydrochalcones	Retrochalcones

**7318**	Licochalcone D	RETRVWFVEFCGOK-RMKNXTFCSA-N	Organic compounds	Phenylpropanoids and polyketides	Linear 1,3-diarylpropanoids	Chalcones and dihydrochalcones	3-prenylated chalcones
**5960**	Isoliquiritigenin	DXDRHHKMWQZJHT-FPYGCLRLSA-N	Organic compounds	Phenylpropanoids and polyketides	Linear 1,3-diarylpropanoids	Chalcones and dihydrochalcones	2'-Hydroxychalcones
**8137**	Glycycoumarin	NZYSZZDSYIBYLC-UHFFFAOYSA-N	Organic compounds	Phenylpropanoids and polyketides	Isoflavonoids	Hydroxyisoflavonoids	Hydroxyisoflavonoids
**10318**	Licoflavone B	GLDVIKFETPAZNV-UHFFFAOYSA-N	Organic compounds	Phenylpropanoids and polyketides	Flavonoids	Flavones	6-prenylated flavones
**10330**	Licoflavone C	MEHHCBRCXIDGKZ-UHFFFAOYSA-N	Organic compounds	Phenylpropanoids and polyketides	Flavonoids	Flavones	8-prenylated flavones
**5980**	Echinatin	QJKMIJNRNRLQSS-WEVVVXLNSA-N	Organic compounds	Phenylpropanoids and polyketides	Linear 1,3-diarylpropanoids	Chalcones and dihydrochalcones	Retrochalcones
**5981**	Licochalcone A	KAZSKMJFUPEHHW-DHZHZOJOSA-N	Organic compounds	Phenylpropanoids and polyketides	Linear 1,3-diarylpropanoids	Chalcones and dihydrochalcones	Retrochalcones
**5982**	Licochalcone B	DRDRYGIIYOPBBZ-XBXARRHUSA-N	Organic compounds	Phenylpropanoids and polyketides	Linear 1,3-diarylpropanoids	Chalcones and dihydrochalcones	Retrochalcones
**5983**	Isoliquiritin	YNWXJFQOCHMPCK-FPYGCLRLSA-N	Organic compounds	Phenylpropanoids and polyketides	Flavonoids	Flavonoid glycosides	Flavonoid O-glycosides
**17774**	Licoflavone B	GLDVIKFETPAZNV-UHFFFAOYSA-N	Organic compounds	Phenylpropanoids and polyketides	Flavonoids	Flavones	6-prenylated flavones
**17777**	Glyinflanin I; (R)-form	AFQCFVZKNRARLS-UHFFFAOYSA-N	Organic compounds	Phenylpropanoids and polyketides	Isoflavonoids	Pyranoisoflavonoids	Pyranoisoflavonoids
**17781**	Glyinflanin E	YSMQGBBJPVUXEX-UHFFFAOYSA-N	Organic compounds	Phenylpropanoids and polyketides	Linear 1,3-diarylpropanoids	Chalcones and dihydrochalcones	Retro-dihydrochalcones
**7162**	Licoflavone A	HJGURBGBPIKRER-UHFFFAOYSA-N	Organic compounds	Phenylpropanoids and polyketides	Flavonoids	Flavones	6-prenylated flavones
**17787**	Inflacoumarin A	RNBLSJGPSGNSIN-UHFFFAOYSA-N	Organic compounds	Phenylpropanoids and polyketides	Neoflavonoids	Prenylated neoflavonoids	Prenylated neoflavonoids

**17791**	Licochalcone D	RETRVWFVEFCGOK-RMKNXTFCSA-N	Organic compounds	Phenylpropanoids and polyketides	Linear 1,3-diarylpropanoids	Chalcones and dihydrochalcones	3-prenylated chalcones
Glycyrrhiza uralensis							
**17793**	Uralenneoside	VWQASRWQZBVNEI-UHFFFAOYSA-N	Organic compounds	Benzenoids	Benzene and substituted derivatives	Benzoic acids and derivatives	p-Hydroxybenzoic acid alkyl esters
**17794**	6''-Acetylliquiritin	HKUBLIRXXFRGKE-UHFFFAOYSA-N	Organic compounds	Phenylpropanoids and polyketides	Flavonoids	Flavonoid glycosides	Flavonoid O-glycosides
**17797**	Kanzonol H	JRVDUBFSQWHYRJ-UHFFFAOYSA-N	Organic compounds	Phenylpropanoids and polyketides	Isoflavonoids	O-methylated isoflavonoids	5-O-methylated isoflavonoids
**17799**	Kanzonol L	CLXMHBYPZWNJQI-UHFFFAOYSA-N	Organic compounds	Phenylpropanoids and polyketides	Isoflavonoids	Isoflavans	6-prenylated isoflavanones
**6280**	2',4,4'-Trihydroxy-3'-prenylchalcone	DUWPGRAKHMEPCM-IZZDOVSWSA-N	Organic compounds	Phenylpropanoids and polyketides	Linear 1,3-diarylpropanoids	Chalcones and dihydrochalcones	3-prenylated chalcones
**17800**	Kanzonol N	ANRYVYXUTFJPOR-UHFFFAOYSA-N	Organic compounds	Phenylpropanoids and polyketides	Isoflavonoids	O-methylated isoflavonoids	5-O-methylated isoflavonoids
**17801**	Kanzonol M	KZQAGRHRFKIERV-UHFFFAOYSA-N	Organic compounds	Phenylpropanoids and polyketides	Isoflavonoids	O-methylated isoflavonoids	5-O-methylated isoflavonoids
**17807**	Kanzonol G	OWVFKLIUBOKWCT-UHFFFAOYSA-N	Organic compounds	Phenylpropanoids and polyketides	Isoflavonoids	Isoflavans	3'-prenylated isoflavanones
**17808**	Kanzonol K	UWUOGPWSIVRQNM-UHFFFAOYSA-N	Organic compounds	Phenylpropanoids and polyketides	Isoflavonoids	Isoflavans	6-prenylated isoflavanones
**17809**	Glyurallin B	DPLWUTYEBRKBLI-UHFFFAOYSA-N	Organic compounds	Phenylpropanoids and polyketides	Isoflavonoids	Isoflav-2-enes	Isoflavones
**17810**	Kanzonol P	ZCGOJWAIXQAUMW-UHFFFAOYSA-N	Organic compounds	Phenylpropanoids and polyketides	Isoflavonoids	Furanoisoflavonoids	Pterocarpans
**7454**	Gancaonin R	QFAPONVNJTUMHF-UHFFFAOYSA-N	Organic compounds	Phenylpropanoids and polyketides	Stilbenes	None	Stilbenes

**7455**	Gancaonin S	VLJOWGMLRJTNDQ-UHFFFAOYSA-N	Organic compounds	Phenylpropanoids and polyketides	Stilbenes	None	Stilbenes
**7456**	Gancaonin U	YJJXCOSDPIJFJR-UHFFFAOYSA-N	Organic compounds	Benzenoids	Phenanthrenes and derivatives	Hydrophenanthrenes	Hydrophenanthrenes
**7457**	Gancaonin V	UEXOPXIMQJMWKA-UHFFFAOYSA-N	Organic compounds	Benzenoids	Phenanthrenes and derivatives	Hydrophenanthrenes	Hydrophenanthrenes
**7205**	Gancaonin C	MEADLGUPYQNUNF-BIIKFXOESA-N	Organic compounds	Phenylpropanoids and polyketides	Isoflavonoids	Isoflav-2-enes	Isoflavones
**7206**	Gancaonin D	UCKSAYIMWMIZQJ-QDEBKDIKSA-N	Organic compounds	Phenylpropanoids and polyketides	Isoflavonoids	O-methylated isoflavonoids	3'-hydroxy,4'-methoxyisoflavonoids
**7208**	Gancaonin L	WSOHPJFMARQRFD-UHFFFAOYSA-N	Organic compounds	Phenylpropanoids and polyketides	Isoflavonoids	Isoflav-2-enes	Isoflavones
**7209**	Gancaonin M	DDLPIQXHEKZHQX-UHFFFAOYSA-N	Organic compounds	Phenylpropanoids and polyketides	Isoflavonoids	O-methylated isoflavonoids	4'-O-methylisoflavones
**7210**	Gancaonin O	AFJYQKPCJLMHCC-UHFFFAOYSA-N	Organic compounds	Phenylpropanoids and polyketides	Flavonoids	Flavones	6-prenylated flavones
**7212**	Gancaonin Q	WGNIVAMNAWBYRO-UHFFFAOYSA-N	Organic compounds	Phenylpropanoids and polyketides	Flavonoids	Flavones	6-prenylated flavones
**8896**	Gancaonin T	OWBYJSNJVWQEQX-UHFFFAOYSA-N	Organic compounds	Phenylpropanoids and polyketides	Stilbenes	None	Stilbenes
**8134**	Glycyrin	FWWGXZYUURXJLK-UHFFFAOYSA-N	Organic compounds	Phenylpropanoids and polyketides	Isoflavonoids	Hydroxyisoflavonoids	Hydroxyisoflavonoids
**5960**	Isoliquiritigenin	DXDRHHKMWQZJHT-FPYGCLRLSA-N	Organic compounds	Phenylpropanoids and polyketides	Linear 1,3-diarylpropanoids	Chalcones and dihydrochalcones	2'-Hydroxychalcones
**8137**	Glycycoumarin	NZYSZZDSYIBYLC-UHFFFAOYSA-N	Organic compounds	Phenylpropanoids and polyketides	Isoflavonoids	Hydroxyisoflavonoids	Hydroxyisoflavonoids
**5980**	Echinatin	QJKMIJNRNRLQSS-WEVVVXLNSA-N	Organic compounds	Phenylpropanoids and polyketides	Linear 1,3-diarylpropanoids	Chalcones and dihydrochalcones	Retrochalcones

**5981**	Licochalcone A	KAZSKMJFUPEHHW-DHZHZOJOSA-N	Organic compounds	Phenylpropanoids and polyketides	Linear 1,3-diarylpropanoids	Chalcones and dihydrochalcones	Retrochalcones
**5982**	Licochalcone B	DRDRYGIIYOPBBZ-XBXARRHUSA-N	Organic compounds	Phenylpropanoids and polyketides	Linear 1,3-diarylpropanoids	Chalcones and dihydrochalcones	Retrochalcones
**5983**	Isoliquiritin	YNWXJFQOCHMPCK-FPYGCLRLSA-N	Organic compounds	Phenylpropanoids and polyketides	Flavonoids	Flavonoid glycosides	Flavonoid O-glycosides
**8542**	1-O-Protocatechuyl-beta-D-xylose	VWQASRWQZBVNEI-UHFFFAOYSA-N	Organic compounds	Benzenoids	Benzene and substituted derivatives	Benzoic acids and derivatives	p-Hydroxybenzoic acid alkyl esters
**5989**	Licoflavonol	TVMHBSODLWMMMV-UHFFFAOYSA-N	Organic compounds	Phenylpropanoids and polyketides	Flavonoids	Flavones	6-prenylated flavones
**5994**	Licoricone	GGWMNTNDTRKETA-UHFFFAOYSA-N	Organic compounds	Phenylpropanoids and polyketides	Isoflavonoids	O-methylated isoflavonoids	4'-O-methylisoflavones
**5996**	Gancaonin A	JQNSUDIGIIGIOL-UHFFFAOYSA-N	Organic compounds	Phenylpropanoids and polyketides	Isoflavonoids	Isoflavans	6-prenylated isoflavanones
**5998**	Gancaonin N	STFVTZQCNYBLNE-UHFFFAOYSA-N	Organic compounds	Phenylpropanoids and polyketides	Isoflavonoids	Isoflavans	6-prenylated isoflavanones
**7162**	Licoflavone A	HJGURBGBPIKRER-UHFFFAOYSA-N	Organic compounds	Phenylpropanoids and polyketides	Flavonoids	Flavones	6-prenylated flavones
**7163**	Lupiwighteone	YGCCASGFIOIXIN-UHFFFAOYSA-N	Organic compounds	Phenylpropanoids and polyketides	Isoflavonoids	Isoflav-2-enes	Isoflavones

Table S9. List of chemicals which are co-targeted on S5R1 and S5R2.

Chemical ID	Chemical Name	inchikey	Chemical Class				
			Kingdom	Superclass	Class	Subclass	Direct_parent
**1925**	Ergost-4-en-3-one	QQIOPZFVTIHASB-UHFFFAOYSA-N	Organic compounds	Lipids and lipid-like molecules	Steroids and steroid derivatives	Ergostane steroids	Ergosterols and derivatives
**1926**	Ergost-4-en-3,6-dione	HYALICAWDSDCPS-UHFFFAOYSA-N	Organic compounds	Lipids and lipid-like molecules	Steroids and steroid derivatives	Ergostane steroids	Ergosterols and derivatives
**1927**	Stigmast-4-en-3,6-dione	UVFOCYGYACXLAY-UHFFFAOYSA-N	Organic compounds	Lipids and lipid-like molecules	Steroids and steroid derivatives	Stigmastanes and derivatives	Stigmastanes and derivatives
**1928**	Stigmasta-4,22-dien-3-one	MKGZDUKUQPPHFM-CMDGGOBGSA-N	Organic compounds	Lipids and lipid-like molecules	Steroids and steroid derivatives	Stigmastanes and derivatives	Stigmastanes and derivatives
**10505**	17-Deoxywithanone	ZTEVDTFJUUJBLP-UHFFFAOYSA-N	Organic compounds	Lipids and lipid-like molecules	Steroids and steroid derivatives	Steroid lactones	Withanolides and derivatives
**1929**	Stigmasta-4,22-dien-3,6-dione	XWHBTBBUPBKDBB-CMDGGOBGSA-N	Organic compounds	Lipids and lipid-like molecules	Steroids and steroid derivatives	Stigmastanes and derivatives	Stigmastanes and derivatives
**10632**	24-Ethyl-24-dehydrocholesterol	YYLFLRIUDMIWTD-UHFFFAOYSA-N	Organic compounds	Lipids and lipid-like molecules	Steroids and steroid derivatives	Stigmastanes and derivatives	Stigmastanes and derivatives
**10636**	Ergostenone(24R)	QQIOPZFVTIHASB-UHFFFAOYSA-N	Organic compounds	Lipids and lipid-like molecules	Steroids and steroid derivatives	Ergostane steroids	Ergosterols and derivatives
**10637**	Stigmasta-4,22-dien-3-one	MKGZDUKUQPPHFM-CMDGGOBGSA-N	Organic compounds	Lipids and lipid-like molecules	Steroids and steroid derivatives	Stigmastanes and derivatives	Stigmastanes and derivatives
**10638**	Stigmasta-4,6-dien-3-one	KEAZWUZFBSXOMV-UHFFFAOYSA-N	Organic compounds	Lipids and lipid-like molecules	Steroids and steroid derivatives	Stigmastanes and derivatives	Stigmastanes and derivatives
**2702**	Castasterone	VYUIKSFYFRVQLF-UHFFFAOYSA-N	Organic compounds	Lipids and lipid-like molecules	Steroids and steroid derivatives	Bile acids, alcohols and derivatives	Tetrahydroxy bile acids, alcohols and derivatives
**9612**	Kusunol	MQWIFDHBNGIVPO-UHFFFAOYSA-N	Organic compounds	Lipids and lipid-like molecules	Prenol lipids	Sesquiterpenoids	Eremophilane, 8,9-secoeremophilane and furoeremophilane sesquiterpenoids
**10633**	Stigmasta-5,24(28)-dien-3-ol	OSELKOCHBMDKEJ-QPSGOUHRSA-N	Organic compounds	Lipids and lipid-like molecules	Steroids and steroid derivatives	Stigmastanes and derivatives	Stigmastanes and derivatives
**10635**	Cholesta-4,6-dien-3-one	XIWMRKFKSRYSIJ-UHFFFAOYSA-N	Organic compounds	Lipids and lipid-like molecules	Steroids and steroid derivatives	Cholestane steroids	Cholesterols and derivatives

**1940**	Isofucosterol	OSELKOCHBMDKEJ-YXSASFKJSA-N	Organic compounds	Lipids and lipid-like molecules	Steroids and steroid derivatives	Stigmastanes and derivatives	Stigmastanes and derivatives
**11158**	Dehydroabietinal	YCLCHPWRGSDZKL-UHFFFAOYSA-N	Organic compounds	Lipids and lipid-like molecules	Prenol lipids	Diterpenoids	Diterpenoids
**2585**	Panax ginseng glycoside P1	DAPLORBFRRJHHY-UHFFFAOYSA-N	Organic compounds	Lipids and lipid-like molecules	Steroids and steroid derivatives	Steroidal glycosides	Steroidal glycosides
**2330**	alpha-Teresantalic acid	QHDPITNBVDSMQH-UHFFFAOYSA-N	Organic compounds	Lipids and lipid-like molecules	Prenol lipids	Monoterpenoids	Bicyclic monoterpenoids
**1956**	31-Norlanost-8-en-3-ol	IXVNEXDHXGHWLS-UHFFFAOYSA-N	Organic compounds	Lipids and lipid-like molecules	Steroids and steroid derivatives	Cholestane steroids	Cholesterols and derivatives
**1959**	31-Norlanost-9(11)-enol	SZCKXGWHINUNKB-UHFFFAOYSA-N	Organic compounds	Lipids and lipid-like molecules	Steroids and steroid derivatives	Cholestane steroids	Cholesterols and derivatives
**2345**	Campesterol	SGNBVLSWZMBQTH-UHFFFAOYSA-N	Organic compounds	Lipids and lipid-like molecules	Steroids and steroid derivatives	Ergostane steroids	Ergosterols and derivatives
**1961**	Lophenol	LMYZQUNLYGJIHI-UHFFFAOYSA-N	Organic compounds	Lipids and lipid-like molecules	Steroids and steroid derivatives	Cholestane steroids	Cholesterols and derivatives
**3118**	Stigmastane-3,6-dione (5alpha H)	HMMVBUVVQLUGQA-UHFFFAOYSA-N	Organic compounds	Lipids and lipid-like molecules	Steroids and steroid derivatives	Stigmastanes and derivatives	Stigmastanes and derivatives
**1967**	4-alpha-Methylcholest-8-en-3-ol	SCEZIHJVTBQOLS-UHFFFAOYSA-N	Organic compounds	Lipids and lipid-like molecules	Steroids and steroid derivatives	Cholestane steroids	Cholesterols and derivatives
**9519**	5-alpha-Stigmast-22-en-3-one	RTLUSWHIKFIQFU-CMDGGOBGSA-N	Organic compounds	Lipids and lipid-like molecules	Steroids and steroid derivatives	Stigmastanes and derivatives	Stigmastanes and derivatives
**9520**	Stigmast-5-en-3-beta-ol-7-one	ICFXJOAKQGDRCT-UHFFFAOYSA-N	Organic compounds	Lipids and lipid-like molecules	Steroids and steroid derivatives	Stigmastanes and derivatives	Stigmastanes and derivatives
**1970**	Withanolide B	ZTEVDTFJUUJBLP-UHFFFAOYSA-N	Organic compounds	Lipids and lipid-like molecules	Steroids and steroid derivatives	Steroid lactones	Withanolides and derivatives
**2226**	Estradiol	VOXZDWNPVJITMN-UHFFFAOYSA-N	Organic compounds	Lipids and lipid-like molecules	Steroids and steroid derivatives	Estrane steroids	Estrogens and derivatives
**9654**	(-)-Ylangene	VLXDPFLIRFYIME-UHFFFAOYSA-N	Organic compounds	Lipids and lipid-like molecules	Prenol lipids	Sesquiterpenoids	Sesquiterpenoids
**1847**	(-)-alpha-Copaene	VLXDPFLIRFYIME-UHFFFAOYSA-N	Organic compounds	Lipids and lipid-like molecules	Prenol lipids	Sesquiterpenoids	Sesquiterpenoids
**11070**	Aromadendrane-4-beta,10-alpha-diol	DWNPMJOWAWGIMM-UHFFFAOYSA-N	Organic compounds	Lipids and lipid-like molecules	Prenol lipids	Sesquiterpenoids	5,10-cycloaromadendrane sesquiterpenoids

**11076**	Neointermedeol	DPQYOKVMVCQHMY-UHFFFAOYSA-N	Organic compound s	Lipids and lipid-like molecules	Prenol lipids	Sesquiterpenoid s	Eudesmane, isoeudesmane or cycloeudesmane sesquiterpenoids
**1863**	3-beta-Hydroxystigmast-5-en-7-one	ICFXJOAKQGDRCT-UHFFFAOYSA-N	Organic compound s	Lipids and lipid-like molecules	Steroids and steroid derivatives	Stigmastanes and derivatives	Stigmastanes and derivatives
**2001**	Stigmastan-3-one (5-alpha)	BVVFRHKBULZQCQ-UHFFFAOYSA-N	Organic compound s	Lipids and lipid-like molecules	Steroids and steroid derivatives	Stigmastanes and derivatives	Stigmastanes and derivatives
**4562**	3,5-Acoradiene	ZQMYLSWMANINAL-UHFFFAOYSA-N	Organic compound s	Hydrocarbon s	Unsaturated hydrocarbon s	Branched unsaturated hydrocarbons	Branched unsaturated hydrocarbons
**3032**	Ledol	AYXPYQRXGNDJFU-UHFFFAOYSA-N	Organic compound s	Lipids and lipid-like molecules	Prenol lipids	Sesquiterpenoid s	5,10-cycloaromadendran e sesquiterpenoids
**4187**	Stigmasterol	HCXVJBMSMIARIN-CMDGGOBGSA-N	Organic compound s	Lipids and lipid-like molecules	Steroids and steroid derivatives	Stigmastanes and derivatives	Stigmastanes and derivatives
**1769**	Stigmast-22-en-3-beta-ol	CSVWWLUMXNHWSU-CMDGGOBGSA-N	Organic compound s	Lipids and lipid-like molecules	Steroids and steroid derivatives	Stigmastanes and derivatives	Stigmastanes and derivatives
**10630**	24-Methylcholest-5,22-dien-3-ol; (3-beta,22E,24R)-form	OILXMJHPFNGGTO-BQYQJAHWSA-N	Organic compound s	Lipids and lipid-like molecules	Steroids and steroid derivatives	Ergostane steroids	Ergosterols and derivatives
**1774**	beta-Sitosterol	KZJWDPNRJALLNS-UHFFFAOYSA-N	Organic compound s	Lipids and lipid-like molecules	Steroids and steroid derivatives	Stigmastanes and derivatives	Stigmastanes and derivatives
**10631**	24-Ethyl-22-trans-dehydrocholesterol ; (3-beta,22E,24R)-form	HCXVJBMSMIARIN-CMDGGOBGSA-N	Organic compound s	Lipids and lipid-like molecules	Steroids and steroid derivatives	Stigmastanes and derivatives	Stigmastanes and derivatives
**1776**	Brassicasterol	OILXMJHPFNGGTO-BQYQJAHWSA-N	Organic compound s	Lipids and lipid-like molecules	Steroids and steroid derivatives	Ergostane steroids	Ergosterols and derivatives
**1777**	Cholesterol	HVYWMOMLDIMFJA-UHFFFAOYSA-N	Organic compound s	Lipids and lipid-like molecules	Steroids and steroid derivatives	Cholestane steroids	Cholesterols and derivatives
**2550**	Campest-5-en-7-one-3-beta-ol	LTLKHSBYMNKWPF-UHFFFAOYSA-N	Organic compound s	Lipids and lipid-like molecules	Steroids and steroid derivatives	Ergostane steroids	Ergosterols and derivatives

Table S10. List of chemicals which are co-targeted on S5R1 and AR.

Chemical ID	Chemical Name	inchikey	Chemical Class				
			Kingdom	Superclass	Class	Subclass	Direct_parent
**1984**	Dihydro-epideoxyarteannuin B	SXDUGGRDNCRRHY-UHFFFAOYSA-N	Organic compounds	Lipids and lipid-like molecules	Prenol lipids	Terpene lactones	Germacranolides and derivatives
**9607**	4-Hydroxy-4-methyl-5-dodecanylcyclopenten-2-one	HWMQXCKSSOGAEL-UHFFFAOYSA-N	Organic compounds	Organic oxygen compounds	Organooxygen compounds	Alcohols and polyols	Tertiary alcohols
**3815**	19(4-3)Abeo-12,14,15-trihydroxy-11-methoxyabiet-4(18),8,11,13-Tetraen-7-one	NHLQPIOUCQBSFL-UHFFFAOYSA-N	Organic compounds	Lipids and lipid-like molecules	Prenol lipids	Diterpenoids	Diterpenoids
**9612**	Kusunol	MQWIFDHBNGIVPO-UHFFFAOYSA-N	Organic compounds	Lipids and lipid-like molecules	Prenol lipids	Sesquiterpenoids	Eremophilane, 8,9-secoeremophilane and furoeremophilane sesquiterpenoids
**1847**	(-)-alpha-Copaene	VLXDPFLIRFYIME-UHFFFAOYSA-N	Organic compounds	Lipids and lipid-like molecules	Prenol lipids	Sesquiterpenoids	Sesquiterpenoids
**10638**	Stigmasta-4,6-dien-3-one	KEAZWUZFBSXOMV-UHFFFAOYSA-N	Organic compounds	Lipids and lipid-like molecules	Steroids and steroid derivatives	Stigmastanes and derivatives	Stigmastanes and derivatives
**2253**	epsilon-Muurolene	NOLWRMQDWRAODO-UHFFFAOYSA-N	Organic compounds	Lipids and lipid-like molecules	Prenol lipids	Sesquiterpenoids	Sesquiterpenoids
**2001**	Stigmastan-3-one (5-alpha)	BVVFRHKBULZQCQ-UHFFFAOYSA-N	Organic compounds	Lipids and lipid-like molecules	Steroids and steroid derivatives	Stigmastanes and derivatives	Stigmastanes and derivatives
**11158**	Dehydroabietinal	YCLCHPWRGSDZKL-UHFFFAOYSA-N	Organic compounds	Lipids and lipid-like molecules	Prenol lipids	Diterpenoids	Diterpenoids
**9654**	(-)-Ylangene	VLXDPFLIRFYIME-UHFFFAOYSA-N	Organic compounds	Lipids and lipid-like molecules	Prenol lipids	Sesquiterpenoids	Sesquiterpenoids

Table S11. List of chemicals which are co-targeted on S5R2 and AR.

			Chemical Class				
**Chemical ID**	**Chemical Name**	**inchikey**	**Kingdom**	**Superclass**	**Class**	**Subclass**	**Direct_parent**
**12777**	Zerumbone	GIHNTRQPEMKFKO-SKTNYSRSSA-N	Organic compounds	Lipids and lipid-like molecules	Prenol lipids	Sesquiterpenoids	Sesquiterpenoids
**9612**	Kusunol	MQWIFDHBNGIVPO-UHFFFAOYSA-N	Organic compounds	Lipids and lipid-like molecules	Prenol lipids	Sesquiterpenoids	Eremophilane, 8,9-secoeremophilane and furoeremophilane sesquiterpenoids
**1847**	(-)-alpha-Copaene	VLXDPFLIRFYIME-UHFFFAOYSA-N	Organic compounds	Lipids and lipid-like molecules	Prenol lipids	Sesquiterpenoids	Sesquiterpenoids
**10638**	Stigmasta-4,6-dien-3-one	KEAZWUZFBSXOMV-UHFFFAOYSA-N	Organic compounds	Lipids and lipid-like molecules	Steroids and steroid derivatives	Stigmastanes and derivatives	Stigmastanes and derivatives
**2001**	Stigmastan-3-one (5-alpha)	BVVFRHKBULZQCQ-UHFFFAOYSA-N	Organic compounds	Lipids and lipid-like molecules	Steroids and steroid derivatives	Stigmastanes and derivatives	Stigmastanes and derivatives
**11158**	Dehydroabietinal	YCLCHPWRGSDZKL-UHFFFAOYSA-N	Organic compounds	Lipids and lipid-like molecules	Prenol lipids	Diterpenoids	Diterpenoids
**9654**	(-)-Ylangene	VLXDPFLIRFYIME-UHFFFAOYSA-N	Organic compounds	Lipids and lipid-like molecules	Prenol lipids	Sesquiterpenoids	Sesquiterpenoids
